# Understanding the public health consequences of suspending a rural syringe services program: a qualitative study of the experiences of people who inject drugs

**DOI:** 10.1186/s12954-019-0305-7

**Published:** 2019-05-21

**Authors:** Sean T. Allen, Suzanne M. Grieb, Allison O’Rourke, Ryan Yoder, Elise Planchet, Rebecca Hamilton White, Susan G. Sherman

**Affiliations:** 1 Department of Health, Behavior and Society, Johns Hopkins Bloomberg School of Public Health, 624 N. Broadway, Baltimore, MD 21205 USA; 2 Center for Child and Community Health Research, Department of Pediatrics, Johns Hopkins School of Medicine, Baltimore, MD 21224 USA; 3 DC Center for AIDS Research, Department of Psychology, George Washington University, 2125 G St. NW, Washington, DC 20052 USA; 4 Department of Health Policy and Management, Johns Hopkins Bloomberg School of Public Health, 624 N. Broadway, Baltimore, MD 21205 USA; 5 Department of Mental Health, Johns Hopkins Bloomberg School of Public Health, 624 N. Broadway, Baltimore, MD 21205 USA

**Keywords:** People who inject drugs, rural drug use, harm reduction, syringe services program, needle exchange

## Abstract

**Background:**

Syringe services programs (SSPs) are evidence-based interventions that are associated with decreases in prevalence and incidence rates of HIV and viral hepatitis among people who inject drugs (PWID). SSPs are also effective conduits to deliver overdose prevention resources among PWID. In December 2015, the Kanawha-Charleston Health Department (KCHD) in West Virginia implemented a SSP; however, the program was indefinitely suspended in early 2018 following policy changes that would have forced the program to operate in ways that conflicted with established best practices. The purpose of this research is to explore the public health implications of the suspension of the KCHD SSP among rural PWID.

**Methods:**

We conducted semi-structured interviews with 27 PWID (59.3% male, 88.9% White) to explore access to sterile injection equipment and overdose prevention resources, high-risk injection practices, and HIV risk perceptions following the KCHD SSP suspension. Participants were recruited from street locations frequented by PWID. Interviews were audio-recorded and transcribed *verbatim*. We employed an iterative, modified constant comparison approach to systematically code and synthesize textual interview data.

**Results:**

Participants described the KCHD SSP as providing a variety of harm reduction services to PWID and being able to speak honestly with SSP staff about their drug use without fear of stigmatization. The suspension of the KCHD SSP fundamentally changed the public health landscape for PWID, ushering in a new era of increased risks for acquiring bloodborne infections and overdose. PWID described more frequently injecting with used syringes and engaging in a range of high-risk injection practices after the SSP was suspended. PWID also discussed having decreased access to naloxone and being less likely to get routinely tested for HIV following the KCHD SSP suspension.

**Conclusions:**

This research demonstrates that the suspension of a SSP in rural West Virginia increased risks for HIV/HCV acquisition and overdose among PWID. The suspension of the SSP led to community-wide decreases in access to sterile injection equipment and naloxone among PWID. The suspension of the KCHD SSP should be viewed as a call to action for sustaining evidence-based interventions in the face of sociopolitical forces that attempt to subvert public health.

## Background

Rural communities throughout the USA need evidence-based interventions to reduce the consequences of the opioid epidemic (e.g., HIV and viral hepatitis outbreaks, overdose fatalities) among people who inject drugs (PWID) [1–4]. Syringe services programs (SSPs) are effective structural interventions that have demonstrated public health benefits [5–13]. Through the provision of sterile injection equipment, SSPs prevent the spread of HIV/HCV infections among PWID, resulting in significant cost savings [14–16]. Additionally, SSPs may offer wraparound services, including HIV/HCV testing, naloxone distribution, and linkages to other services [17]. Although there is consensus in the literature regarding the benefits of SSPs, most studies examined their implementation in urban areas [18]. This creates an important gap in the literature given the ongoing vulnerability for HIV/HCV outbreaks among PWID in rural communities [19, 20].

Rural areas pose unique obstacles for implementing evidence-based public health strategies (e.g., SSPs), including limited transportation options, lack of anonymity, diminished access to HIV/HCV testing, and stigmatization of drug use [21–27]. However, the enabling environment for rural SSP implementation has evolved in recent years; for example, some rural communities have taken legal steps to implement SSPs (e.g., decriminalizing syringe possession). These activities stem from the Scott County HIV outbreak and the US Congress lifting the ban on using Federal monies to support SSP operations [1, 19, 20, 28, 29]. In response to the opioid epidemic, the Kanawha-Charleston Health Department (KCHD) in West Virginia launched a single, fixed-site SSP in December 2015 that was located within the health department facility [30, 31]. The KCHD SSP provided access to sterile injection equipment, hepatitis testing and vaccinations, testing for HIV and other sexually transmitted infections, wound assessments, flu shots, contraceptives, drug treatment referrals, and overdose prevention resources. Although the program initially provided services to a small number of PWID, by 2018, it served more than 400 PWID each week [30]. This level of service delivery was remarkable given the relative rurality of Kanawha County; although Kanawha County contains the city of Charleston, 90.3% of its land space is characterized as rural by the US Census Bureau [32].

The volume of service delivery at the KCHD SSP was commendable by public health standards, but, like urban areas [33], it was not free from “not in my backyard” or NIMBY-based opposition. In early 2018, the program was implicated as precipitating increases in discarded syringes and crime [34–36]. Community-level division about the SSP was also exacerbated by sociopolitical forces leading up to the November 2018 election in which the outgoing-Mayor was a vocal opponent of the program and the then-Mayoral candidates had conflicting views on how to best meet the public health needs of PWID [37]. Opposition to the KCHD SSP culminated in law enforcement implementing a number of operational restrictions on the program that were not considered best practice, such as restricting services to only those PWID who could prove county residence, mandating testing for bloodborne infections, only distributing retractable syringes, and requiring participants to present government-issued photo identifications [38]. Faced with operating under rules that were demonstrably not best practice, KCHD suspended SSP operations in March 2018 and later announced the program was suspended indefinitely [36, 39]. The suspension of the KCHD SSP presents a unique opportunity to advance our understanding of the public health implications of suspending rural SSP operations. Given the expansion of SSP services in non-urban areas, this research is both timely and responsive to the modern era of NIMBY-based opposition to rural SSP operations. The purpose of this research is to explore the public health implications of the suspension of the KCHD SSP.

## Methods

We conducted in-depth interviews with PWID in Charleston (Kanawha County), West Virginia, in September 2018. Eligible participants were current residents of Kanawha County, West Virginia, aged 18 years or over who had injected illicit drugs in the past 30 days. Participants were recruited from locations frequented by PWID. Given the sensitive nature of this study, all data were collected anonymously.

Once eligibility and interest were determined, participants were invited to complete the 45–75-min in-depth interview. The interview followed a semi-structured format. After collecting demographic information, we asked participants about their experiences with the KCHD SSP when it was operational, their access to sterile syringes and overdose prevention resources after the SSP suspension, syringe sharing, perceptions of HIV risk, and current challenges to HIV prevention. Participants were compensated $40 for their time. Interviews were audio-recorded with participants’ permission and transcribed *verbatim*. All study procedures were approved by the Johns Hopkins School of Public Health Institutional Review Board.

Data analysis was conducted using an iterative, constant comparative approach [40, 41]. The lead qualitative researcher read through 10 transcripts to develop the initial coding framework via open coding. Multiple iterations of the codebook were created through coding of additional transcripts and subsequent reflection and discussion by three qualitative coders and the other co-authors. The three coders then independently applied the codes systematically to a cross-section of transcripts, and the reliability between coders was tested. According to conventions on the use of Kappa for interrater agreement, the consistency between coders was satisfactory (≥ 0.70) [42, 43], and the coders independently coded the remaining transcripts in Atlas.ti software. Thematic codes were compared within a single interview and between interviews, and variability was considered based on participants’ gender [40, 41]. Qualitative data are presented in this article using direct quotations from the participants to illustrate findings.

## Results

Twenty-seven participants completed in-depth interviews (16 males, 11 females). Participant characteristics are presented in Table 1. The majority were White (88.9%) and homeless (81.5%). Participants primarily preferred injecting crystal methamphetamine (59.3%) or heroin (40.7%). Among our participants, 77.8% (*n* = 21) directly accessed services at the KCHD SSP while it was operational. Participants who did not directly access services at the KCHD SSP reported several reasons for not doing so, including because they acquired sterile injection equipment from others (e.g., PWID who directly accessed KCHD SSP services or family/friends that had diabetes and therefore had access to sterile syringes). Our discussions about the suspension of the KCHD SSP centered around six domains: community-level stigmatization of PWID, experiences at the KCHD SSP when it was operational, access to sterile injection equipment following the KCHD SSP suspension, risk behaviors associated with not having access to sterile syringes, HIV risk perceptions following the SSP suspension, and naloxone access after the SSP was suspended.

**Table 1:** Sociodemographic characteristics and drug use among people who inject drugs (*N* = 27) in Charleston, West Virginia

	Male *n* (%)	Female *n* (%)	Total *n* (%)
Age, median (IQR)	37 (11.5)	40 (15)	38 (13)
Race			
White	14 (87.5)	10 (90.9)	24 (88.9)
Black/African American	2 (12.5)	1 (0.9)	3 (11.1)
Housing			
Homeless	14 (87.5)	8 (72.7)	22 (81.5)
Unstably housed	1 (6.3)	2 (18.2)	3 (11.1)
Stably housed	1 (6.3)	1 (9.1)	2 (7.4)
Preferred injection drug^a^			
Heroin	6 (37.5)	5 (45.5)	11 (40.7)
Suboxone	5 (31.3)	2 (18.2)	7 (25.9)
Speedball (heroin and cocaine)	0 (0.0)	1 (9.1)	1 (3.7)
Crystal Methamphetamine	11 (68.8)	5 (45.5)	16 (59.3)
Previous client of the KCHD SSP			
Yes	12 (75.0)	9 (81.8)	21 (77.8)
No	4 (25.0)	2 (18.2)	6 (22.2)

^a^Participants could identify more than one preferred drug

### Community-level stigmatization of PWID

Participants described being stigmatized for their drug use before, during, and after KCHD SSP operations, and that stigmatization served as a disincentive for accessing health and human services. As noted by a 38-year-old male, “I want to change, but it’s hard to change when they make you feel different than or less than—.” Another participant explained the broader community perception of PWID as, “…they think we’re the most disgusting, evil, cold-hearted people they’ve ever seen…” (28-year-old male). Participants described the residents of Charleston as both lacking empathy for people with substance use disorders and devaluing the lives of PWID:

[PWID] must be uneducated and stupid and dirty and homeless and just worthless. And that’s not the case. I mean, there are IV drug users in every aspect of life. I mean, I’ve known lawyers and doctors that, you know, do the same thing that I do [inject drugs]. You know, I have a college degree. I have-- I’ve always had a good job, my entire life, until last year. The last year I’ve had some problems, but I’m far from uneducated, and I’m far from, you know, dirty or someone to be thrown away and just, you know, forgotten about. But a lot of people look at it that way. (41-year-old female)

Participants also explained that stigmatization of drug use manifested on a Facebook group in which community members posted pictures of individuals they perceived to be PWID, “I mean, around here in Charleston, they hate us. And they know us. They take pictures of us…” (38-year-old male). Participants linked this exploitative behavior to increased likelihoods for adverse health consequences: “It could make somebody so emotional that they go out and try to overdose or anything” (28-year-old male)*.* Further, one participant described her impression of the local community's callous disregard toward the health and wellbeing of PWID:

But a lot of them don’t care if you die. I heard one say, “You know, I’m fooling around with you dope users when I could be out here, helping somebody that needs help, having a hard time.” And I thought that was terrible. We’re all people, you know… And they look at you like you're trash. Like you have a motive in mind for everything. And you don’t. I don’t. I don’t want to be out here, I’m just trying to better myself. (57-year-old female)

### Experiences at the KCHD SSP

Overall, participants described both liking the KCHD SSP and that it provided valuable resources to PWID. As explained by a male participant:

They didn’t judge nobody for their problems and stuff like that. You know, they gave you the syringes to use, you know. They gave you alcohol. They gave you sharps containers to throw them away in. You know, they helped you in every way they could.

Participants described trusting the staff and being able to talk openly with them about drug use without fear of stigmatization. A 41-year-old female participant, for example, stated:

They [KCHD SSP staff] were always very open to listening to anything we had to say. They would always ask questions. They were just-- and when they were asking questions, they weren’t asking like in a nosy sort of way or anything. They were asking because they wanted to know. They wanted to learn. They wanted to understand. And I mean, they treated us, you know, in no way, shape, or form, like we were any less. You know, it was a really good program. I was surprised whenever they completely done away with it, I really was*.*

As explained by another female participant, “They taught me not to be ashamed to ask for help.” Participants expanded on the destigmatizing nature of service provision at the KCHD SSP by noting the professional manner in which services were provided:

Participant: They were great people. All of them. Very kind. Very nonjudgmental. Very down to earth. And very professional.

Interviewer: What did they do that you liked?

Participant: They made me feel like I was a person. (40-year-old male)

### Accessing sterile injection equipment following the KCHD SSP suspension

Overwhelmingly, participants reported experiencing great difficulties in acquiring sterile injection equipment following the KCHD SSP suspension. As explained by a 29-year-old male, “So now, it’s making it damn near impossible to get them [sterile syringes]. So people’s, you know, they’re like gold out here now. There’s not very many of them.” Participants identified seven methods for accessing sterile syringes in the post-KCHD SSP era: buying syringes on the street, purchasing syringes at pharmacies, purchasing syringes online, stealing syringes from emergency departments, stealing syringes from someone with diabetes, receiving syringes from family or friends that live elsewhere and have access to a SSP, and accessing syringes at a SSP that operates at a nonprofit clinic in Charleston. Except for buying syringes on the street, participants reported engaging in these methods to acquire sterile injection equipment infrequently. After the KCHD SSP suspension, most participants reported purchasing sterile syringes on the street at the cost of $1–$6 (USD) each. Participants noted that the PWID selling syringes had access to them because they or someone they know is diabetic and has consistent access to sterile syringes. PWID also described the availability of syringes on the streets as being unstable and varying by time; for example, a 39-year-old participant explained:

Since they closed down the needle exchange it’s kind of hard [to find sterile syringes]. But, you know, I do know people that get some, and people that’s diabetic- they’ll sell their needles too, so, yeah. And sometimes it’s hard, it just depends on what time of the month it is. Sometimes it’s hard and then sometimes I can go buy a whole bag…

A small number of participants were aware that sterile syringes could be accessed at a nonprofit clinic-based SSP in Charleston; however, only five reported having gone to the clinic for harm reduction services. Among those five participants, none had recently accessed sterile injection equipment at the clinic SSP and most reported having only gone once for harm reduction services. Participants described experiencing a range of adverse experiences at the clinic, both in terms of its SSP and broader healthcare services; for example, a female participant described the staff at the clinic as stigmatizing:

They’re not accepting and they’re just very rude. They’re very rude with me and I don’t like going there at all. And I had been, like I said, I went to the exchange program there the one time and I haven’t been back. You know, they just make you feel really uncomfortable. They make you feel like you’re a bad person, like, you know what I mean?

This participant also noted that the staff at the clinic SSP made them feel as though they were a “bad” person for being addicted to drugs and that their program was in stark contrast to the KCHD SSP:

I mean, they act like it’s like a, I don’t know, kind of like I’m a-- like I need to stand out like I’m a kind of like a bad person just because I’m coming in for the exchange. And just I mean, it makes you feel uncomfortable, really, you know. It’s not like it was at the Health Department at all.

Additionally, participants reported not going to the clinic SSP because they distributed retractable syringes; for example, a male participant explained:

The ones [syringes] at the [clinic SSP] are retractable, one-time use. And it’s like, well, you cannot push them down all the way but then you’re not getting all your dope or… I mean, I’ve figured out where I can actually take the syringe apart and put it back together where it will retract but you know, that can be an ordeal sometimes especially when you’re on the streets. You don’t want it to get like contaminated or whatever.

Another male participant succinctly described their rationale for not utilizing the nonprofit clinic SSP as, “That’s what they give [retractable syringes] at [clinic SSP] now, and that’s one of the reasons-- I haven’t went down there.”

### Risk behaviors associated with the lack of sterile syringes

Participants described the suspension of the KCHD SSP as precipitating a scarcity of sterile injection equipment among the PWID population, resulting in high-risk injection drug use-associated scenarios. Among our participants, the majority reported not wanting to share syringes because of risks for HIV and viral hepatitis acquisition; however, only a few described being able to avoid this behavior when sterile syringes were unavailable. To avoid syringe sharing, some participants reused their syringes for as long as possible:

If I can’t find [sterile syringes] -- I’ll just use the same one that I’ve got. But right now, like I say I’ve got one that the needle’s actually turning on it and bent up and stuff. So yeah… I mean, it hurts really bad. It strikes a nerve in you and honestly it leaves worse-- I mean worse marks. A new one wouldn’t do all that right there < points to tract marks and abscesses on arm>. (28-year-old male)

A small number of participants reported using drugs via other routes of administration (e.g., smoking or snorting) to avoid syringe sharing. Most frequently, however, participants described injecting with used syringes when unable to acquire a sterile syringe. A 36-year-old man described the post-KCHD SSP period as a “[needle exchange] from that homeless person to that homeless person to that homeless person to that homeless person.” Many of the participants reported attempting to reduce their risks for bloodborne infections via restricting who they share syringes with to close friends and sexual partners. A 24-year-old woman, for example, described only sharing syringes with four of her female friends:

I’m not just going to share with anybody, but I know-- there’s about four of us. We always come to each other because we know that if this one doesn’t have it [a syringe] and this one doesn’t have it, and this one doesn’t, then they’ll have it.

A small number of PWID also described disclosing their HIV and hepatitis serostatus with whom they were sharing syringes in attempts to reduce risks: “I know what I have, and I let them know what I have, like ‘I have hep C, so you use that [syringe] you’re 99.9 percent assured of getting hep C unless you’-- but most of us have it, so…” (24-year-old female).

In the absence of the KCHD SSP, participants also described purchasing “gently used” injection equipment on the street:

And most of the time if you can’t find that person who has got some clean ones [syringes], man, you’re just trying to take the first rig you can get to like, ‘I got this one, man. I only used three times. But it’s practically new, you know what I’m saying?’ By that time you want it so bad, you’re like, man, I don’t care. I’ll give you 10 bucks for that needle, just give it to me, man. (35-year-old male)

Finally, participants described injecting with used syringes that had been discarded by others (e.g., on the street):

…and I have found needles and I have went and bleached them, which I know darn well that that was disgusting…but I pick them up off of the street and cleaned them and bleached them and used them. (38-year-old male)

Notably, participants described a nexus of high-risk syringe sharing behaviors in the absence of the KCHD SSP; for example:

It’s an epidemic [syringe sharing]. That’s how bad it’s getting. I mean, you’ve got four or five people sharing a needle, and then they’ll throw it down on the ground and maybe somebody else come along and they pick it up and they think burning it with a lighter will sterilize it. Well, that’s not so. And then the next thing you know, four or five people use that needle. One needle will probably do 15 people. (49-year-old male)

### HIV risk perceptions following The KCHD SSP suspension

When asked about their HIV risk, female participants primarily described their risk in relation to having to inject with used syringes in the absence of the KCHD SSP. Without the program, female participants perceived their HIV risk as being greater than when the SSP was operational due to decreased availability of sterile injection equipment. A 24-year-old female participant described this, stating:

It sucks that we don’t have a needle exchange, and, yeah, pretty much we all say that if you don’t have Hep C then you’re the police, because we’ve all had to share something at some point… it’s gross. It grosses me out. Every time I do a shot I go ‘I feel it. I feel the HIV coursing through my veins.’ And it’s a joke, but at the same time, it’s not. I laugh about it because if I don’t, I’d probably scream, but it’s not good for all of us to be out there doing that, using [after] each other.

Another female participant explained:

Now I’m so worried about contracting HIV, it’s unreal. I’ve been tested four times already this year…I know a couple somebodies that have HIV. And I’m really afraid that I’m going to contract it. I know that this person lets other people borrow needles, and I’m afraid I am going to get the same one [syringe] they’ve used. It scares me to death.

Male participants associated syringe sharing with elevated risks for HIV acquisition and described having to inject with used syringes more frequently following the suspension of the KCHD SSP. However, unlike their female counterparts, male participants did not view their personal risks for HIV acquisition as having increased since the KCHD SSP suspension. Male participants suggested that occasionally injecting with a used syringe did not greatly affect their HIV risk. A 35-year-old man demonstrates this, saying:

[My HIV risk is] minimal. Because I don’t really buy many used needles or share needles with people. Like the last five or six months I’ve probably bought four used needles. I probably shared twice with someone. Those are the people that I was already sleeping with anyway so it’s like I’m already having sex with you and I’m protected. So, I’ll use your needle. It’s your blood. We’re still doing it anyway.

Similarly, a 25-year-old man who described sharing syringes with acquaintances also felt his HIV risk was low because he was confident in his ability to discern if someone was living with HIV and then choose not to share syringes with that individual:

I mean, [the HIV risk is] not nearly as bad as the Hepatitis C risk around here, but, you know, I suppose there’s always a chance of getting it. I mean, there is a risk, but not a high risk, you know?… There’s not as many people with HIV, and those who do have HIV, it’s usually general knowledge around the community.

Related to HIV risk perceptions, participants also described the KCHD SSP as a common location to get tested for HIV and that the suspension of the program presented challenges for routine testing. While most participants knew of other venues to get an HIV test, they reported being less likely to get consistently tested for HIV as doing so became an additional task they had to seek out at venues they were not already otherwise accessing. In essence, the fact that PWID accessed harm reduction services at the KCHD SSP and were simultaneously offered HIV testing increased HIV testing frequency. PWID also described the staff at non-KCHD SSP locations as stigmatizing drug use; for example, a 40-year-old male participant reported:

The only time I ever got tested was from the [KCHD] needle exchange. That is the truth. It’s not because it hadn’t been offered, though. I think in hospitals I didn’t want to [get HIV tested] because they judge you. These hospitals will treat addicts-- I don’t mean to badmouth them because I know they have hard jobs, some of them. But are they all-- This is the problem. I haven’t met a nurse or an assistant or any of them that hasn’t treated me like a drug addict. Whether I’m red-flagged or not, I still deserve the same care, the same respect as anyone else as far as I’m concerned. And if they’d really do their job, they would understand this disease [addiction].

### Naloxone access without the KCHD SSP

Among our participants, the KCHD SSP was commonly reported as a primary source for overdose prevention resources, including naloxone. A 39-year-old woman, for example, shared that “I did attend the Narcan program and I got that [naloxone] about every Wednesday, too. You know, I’ve been witness to about 12 overdoses now and I’ve had to help with CPR and get the Narcan and stuff and it’s really, really helpful.”

With the SSP suspension, however, participants repeatedly stated that they perceived naloxone as not being as accessible and that fewer people are carrying it. Described by a 24-year-old woman: “Since the exchange closed? I thought it [naloxone] disappeared into thin air or something because I have not seen not a one…Oh it’s been months. Months.” A 29-year-old man similarly said, “You can’t get them [naloxone] no more…Don’t nobody got it no more. You won’t see nobody with any of them now.” Many of the participants assumed that the KCHD naloxone program ceased operations along with the SSP, and very few were aware of other ways to access it. Several participants identified that not regularly going to the health department for syringe access was associated with decreased naloxone access as it was an ancillary service they received while interacting with the SSP. As a result, participants overwhelmingly believed that naloxone is not as prevalent in the community and that PWID are more likely to overdose.

## Discussion

The KCHD SSP was widely viewed among PWID as a trustworthy harm reduction program that provided infectious disease and overdose prevention services in a destigmatizing manner. However, the suspension of the program fundamentally changed the public health landscape for PWID, ushering in a new era of increased risks for bloodborne infections and overdose. PWID reported more frequently injecting with used syringes and engaging in a range of high-risk behaviors after the suspension of the KCHD SSP. PWID also perceived decreased availability of naloxone in the post-KCHD SSP era. These data demonstrate that when sociopolitical forces subvert evidence-based programs, the most vulnerable suffer.

Existing literature has demonstrated that SSP implementation is associated with decreases in prevalence and incidence rates of HIV and viral hepatitis among PWID, resulting in significant cost savings [14, 15]. These gains in infectious disease prevention occur via reductions in high-risk injection practices through the provision of sterile injection equipment. Among our sample, PWID reported not wanting to share injection equipment due to associated HIV risks; however, syringe sharing was a practice that was described as occurring with greater frequency following the suspension of the KCHD SSP. Additionally, we found that men and women perceived their risks for HIV acquisition differently (i.e., despite engaging in syringe sharing more frequently following the KCHD SSP suspension, men did not view their personal risks for HIV acquisition as having increased since the suspension). These data serve as a call to action for policymakers to take a stand against inaccurate and misleading reports about SSPs and enact immediate plans to ensure PWID have access to sterile injection equipment and overdose prevention resources. Choosing to ignore the evidence-base for SSP operations not only presents an ethical and moral dilemma, but also sets the stage for an HIV outbreak and worsening overdose epidemic.

Collectively, participants described experiencing pervasive community-level stigmatization of drug use and that it served as a disincentive for seeking health and human services. This finding, while disturbing, is not surprising given that existing research has shown that stigma can negatively affect positive health-seeking behaviors among PWID [44]. Further, the sociopolitical climate leading up to the suspension of the KCHD SSP was characterized by fear-based, inaccurate, and stigmatizing messaging surrounding addiction. For example, the former Chief of Police stated in an interview that officers who were stuck with a syringe could not kiss anyone for a year, a statement that is demonstrably false [45]. The former Mayor of Charleston also described the KCHD SSP as a driver of increased crime: “It was a needle mill. And crime in this city skyrocketed” [34]. Future work should be done to better understand how to dispel community-level stigma toward PWID and inaccurate, fear-based messaging surrounding addiction. Our findings also underscore the importance of educating elected officials about the evidence-base for harm reduction services.

This study fills an important gap in the literature by enhancing our understanding of the public health implications of suspending a SSP that primarily served a rural PWID population. Additionally, our research gives a voice to PWID in a city where they were excluded from evaluations of the KCHD SSP [46]. Our participants’ voice is a step forward for public health in West Virginia as an evaluation of the KCHD SSP put forth by the West Virginia Department of Health and Human Resources did not include the perspective of a single participant [46]. While the KCHD SSP was not perfect [47], objective and balanced assessments of its operations should have, at a minimum, included the voices of those PWID who used the program.

It is important to interpret our findings within the broader community context of Charleston, West Virginia. After the KCHD SSP ceased operations, PWID reported attempting to access services and sterile injection equipment through a variety of other channels. However, these venues and strategies presented challenges that made sustained utilization of sterile injection equipment unlikely. For example, PWID described the SSP at a nonprofit clinic as not only stigmatizing, but also distributing retractable syringes. The distribution of retractable syringes has been deemed “inappropriate for controlling epidemics of HIV, hepatitis C, and other bloodborne viruses among people who inject drugs” [48] and is not considered best practice for SSP operations [49]. Further, the clinic-based SSP has strict operational policies that may create barriers for sustained harm reduction service utilization; for instance, staff at this program described their syringe return policy as, “If you come back without every needle, you will not get any more needles” and that “There are no second chances” [50]. This high threshold for continued access to harm reduction services is counter to public health and not reflective of evidence-based, best practices for syringe services programs. Existing service providers should take swift action to align their operations with best practices and enact immediate efforts to ensure all staff understand the realities of addiction and not stigmatize PWID. Further, policymakers should revise all existing policies surrounding the provision of harm reduction services such that they conform to established best practices for SSP operations [49].

This research is not without limitations; for example, we recruited participants and conducted all interviews in the city of Charleston, West Virginia, but not from more rural areas of Kanawha County. It is possible that the impacts of the KCHD SSP suspension could vary geographically. That said, we would expect those PWID in more distal locations relative to the KCHD to be more adversely affected by the suspension. Another limitation of this research is that most of our participants were White; thus, limiting our ability to evaluate differences between racial groups. Lastly, six of our participants did not access services at the KCHD SSP. While they were not able to speak directly to KCHD SSP operations, they were able to describe the HIV/HCV and overdose prevention landscape while the program was operational and how they indirectly benefitted from its services (e.g., community-level access to sterile injection equipment). Despite these limitations, our research was characterized by several strengths. We were able to learn first-hand about KCHD SSP operations and the impact of the program suspension from a group of PWID that had long histories of lived-experiences in Charleston. Additionally, we were able to access a sample of PWID that reported diverse injection drug use profiles, enhancing the representativeness of our findings.

In conclusion, this research demonstrates that the suspension of a SSP in rural West Virginia led to an era of increased risks for HIV/HCV acquisition and overdose among PWID. The suspension of the SSP led to community-wide decreases in access to sterile injection equipment and naloxone. Sociopolitical forces surrounding KCHD SSP operations cultivated pervasive community-level stigma and served as a disincentive for PWID to pursue positive health-seeking behaviors. This research serves as a call to action for policymakers to not only learn about the realities of addiction and evidence-based programs for PWID, but also vocally defend them in the face of fear-based, inaccurate, and stigmatizing messaging by those who attempt to subvert public health.

## Abbreviations

HCV: Hepatitis C
HIV: Human immunodeficiency virus
IDU: Injection drug use
KCHD: Kanawha-Charleston Health Department
PWID: People who inject drugs
SSP: Syringe services program
US: United States of America
WV: West Virginia
